# Anti-Complementary Components of *Helicteres angustifolia*

**DOI:** 10.3390/molecules21111506

**Published:** 2016-11-10

**Authors:** Xiang Yin, Yan Lu, Zhi-Hong Cheng, Dao-Feng Chen

**Affiliations:** 1 State Key Laboratory of Natural Medicines, China Pharmaceutical University, Nanjing 210009, China; yinxiang2zhy@163.com; 2 Department of Pharmacognosy, School of Pharmacy, Fudan University, Shanghai 201203, China; luyan@fudan.edu.cn (Y.L.); chengzhh@fudan.edu.cn (Z.-H.C.)

**Keywords:** *Helicteres angustifolia*, sterculiaceae, phenalenone, sesquilignan, anti-complement

## Abstract

A first phenalenon derivative with an acetyl side chain at C-8, 8-acetyl-9-hydroxy-3-methoxy-7-methyl-1-phenalenon (compound **1**), and a pair of new sesquilignan epimers at C-7″ of hedyotol C and hedyotol D analogs, hedyotol C 7″-*O-*β-d-glucopyranoside (compound **2**) and hedyotol D 7″-*O*-β-d-glucopyranoside (compound **3**) were isolated from the aerial parts of *Helicteres angustifolia* together with nine known compounds (**4**–**12**). Their structures were elucidated on the basis of spectroscopic methods, including mass spectroscopy, and 1D and 2D nuclear magnetic resonance. Eleven isolates exhibited anti-complementary activity. In particular, compounds **4** and **5** exhibited potent anti-complementary activities against the classical and alternative pathways with CH_50_ values of 0.040 ± 0.009 and 0.009 ± 0.002 mM, and AP_50_ values of 0.105 ± 0.015 and 0.021 ± 0.003 mM, respectively. The targets of compounds **4** and **5** in the complement activation cascade were also identified. In conclusion, the anti-complementary components of *H. angustifolia* possessed chemical diversity and consisted mostly of flavonoids and lignans in this study.

## 1. Introduction

The complement system, as a chief component of innate immunity, plays a significant role in host immune defense against infection and in the clearance of antigen-antibody complexes from the bloodstream. It can be activated by a cascade mechanism through the classical pathway (CP), alternative pathway (AP) or the lectin pathway [1]. Generally, the normal activation of the complement system leads to elimination of invading pathogens and optimal host response. However, excessive activation of the complement system may induce tissue damage, inflammation and a number of pathological situations such as systemic lupus erythematosus, rheumatoid arthritis and acute respiratory distress syndrome [2,3]. Thus, inhibition of the complement system is potentially therapeutic in diseases resulting from uncontrolled or overshooting complement activation. The desirable properties of a valuable anti-complementary therapeutic agent are that it should be inexpensive and highly specific, either having a long plasma half-life or being active orally [2]. However, none of the currently available agents meet all these criteria. A wide array of specific complement inhibitors, for instance cobra venom factor (CVF), synthetic nafamastat mesilate (FUT-175), a recombinant humanized antibody fragment, and a monoclonal antibody, Eculizumab, have been developed to target various components of the complement cascade [4,5,6,7,8]. However, the clinical development of this plethora of complement therapeutics has exhibited some side effects. Therefore, there is an urgent need to search for naturally occurring and low-toxicity anti-complementary agents from traditional Chinese medicines.

The dried roots or whole plants of *Helicteres angustifolia* L. (Sterculiaceae) have been used in Chinese folk medicine as anti-inflammatory, antidotal, analgesic, anti-bacterial and anti-cancer agents [9]. Previous phytochemical studies revealed that this plant mainly contains triterpenoids, penylpropanoids, quinones, lignans and flavonoids [10,11,12]. It is commonly accepted that the complement system is a crucial trigger for inflammation [13,14]. Therefore, one may hypothesize that complement inhibition intercepts the process of complement-dependent inflammation diseases. In our effort to search for anti-complementary agents from Chinese herb medicines and to clarify their anti-complementary constituents [3,15,16], an ethanolic extract of the aerial parts of *H. angustifolia* was found to show a potent anti-complementary activity, which encouraged us to investigate its anti-complementary constituents systematically. In this study, one new skeleton compound (**1**) and two new compounds (**2** and **3**) were isolated from the aerial parts of this plant, together with nine known compounds, on the basis of MS, 1D and 2D NMR, as well as comparison with the literature. Herein, the isolation, characterization, and anti-complementary activity of these compounds are reported. In addition, the target identification in the complement activation cascade has been investigated for anti-complementary constituents.

## 2. Results and Discussion

In our search for anti-complementary activity compounds from plants, the 95% ethanolic extract of *H. angustifolia* possessed a potent anti-complementary activity. Thus, the EtOAc-soluble fraction was concentrated under reduced pressure to produce a residue that was subjected to multiple chromatographies. From this active fraction, 12 compounds (**1**–**12**) including one new skeleton compound (**1**) and two new compounds (**2** and **3**) were isolated and identified.

**Figure 1:**
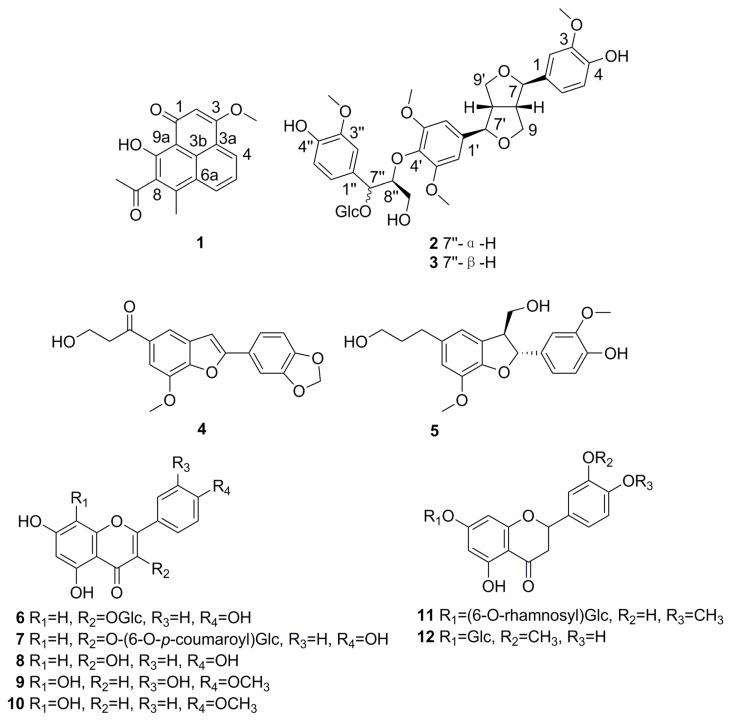
Structures of compounds **1**–**12**.

**Figure 2:**
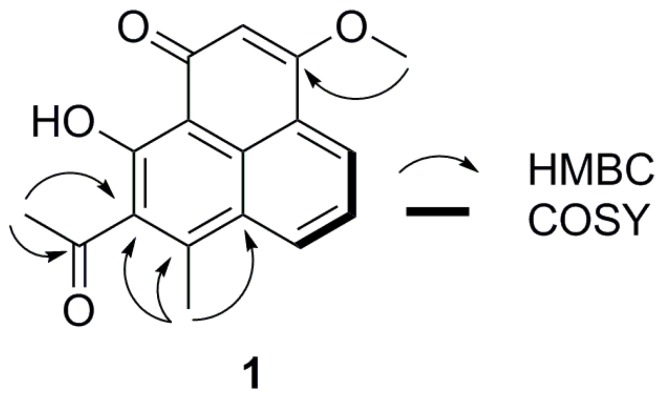
Key HMBC and ^1^H-^1^H COSY correlations for compound **1**.

Compound **1** (Figure 1) was obtained as yellow crystals (CHCl_3_), and its molecular formula was determined as C_17_H_14_O_4_ from HR-ESI-MS (*m*/*z* 283.0962 [M + H]^+^, calcd. for C_17_H_15_O_4_^+^, 283.0965). The ^1^H-NMR spectrum (Table 1) exhibited signals for three aromatic protons (δ_H_ 8.43 (1H, d, *J* = 7.8 Hz) 8.24 (1H, d, *J* = 7.8 Hz) and 7.58 (1H, t, *J* = 7.8 Hz)), an olefinic methine (δ_H_ 6.66 (1H, s)), two methyls (δ_H_ 2.72 and 2.58 (each 3H, s)), a methoxyl (δ_H_ 3.92 (3H, s)) and a *peri*-hydroxy (δ_H_ 17.36 (1H, s)), as well as corresponding with ^13^C-NMR resonances at δ_C_ 127.9, 130.8, 123.7, 100.2, 32.1, 16.0, 56.4 and 174.2, respectively. Furthermore, the ^13^C-NMR spectrum (Table 1) showed two carbonyl carbon signals (δ_C_ 204.1 and 181.1) and eight quaternary carbon signals. The ^1^H-^1^H COSY spectrum revealed the presence of a subunit C-4–C-5–C-6 (Figure 2). These NMR signals indicated that compound **1** had a phenalenone skeleton as in the known compounds 7-methyl-1-phenalenon [17] and myeloconone A_2_ [18]. The HMBC (Figure 2) correlations from 7-CH_3_ (δ_H_ 2.58) to C-7 (δ_C_ 144.1), C-8 (δ_C_ 136.3) and C-6a (δ_C_ 125.4) indicated that the methyl was attached to C-7. Different from 7-methyl-1-phenalenon, compound **1** possessed a methoxyl, an acetyl and a *peri*-hydroxy group in its structure. The HMBC (Figure 2 and see Supplementary Materials) correlations from 3-OCH_3_ (δ_H_ 3.92) to C-3 (δ_C_ 166.6), 8-COCH_3_ (δ_H_ 2.72) to 8-COCH_3_ (δ_C_ 204.1) and C-8 (δ_C_ 136.3) further confirmed that the methoxyl was connected to C-3, and the acetyl group was connected to C-8. Consequently, the structure of **1** was deduced as 8-acetyl-9-hydroxy-3-methoxy-7-methyl-1-phenalenon. To our knowledge, **1** is the first phenalenon derivative with an acetyl side chain connected to C-8.

Compound **2** (Figure 1) was obtained as white amorphous powder, and the molecular formula was determined as C_37_H_46_O_16_ by HR-ESI-MS (*m*/*z* 769.2662 [M + Na]^+^, calcd. for C_37_H_46_NaO_16_^+^, 769.2678). The ^1^H- and ^13^C-NMR spectra (Table 2) showed characteristic signals for a sesquilignan similar to hedyotol C [19], including eight aromatic protons (two sets of 1,2,4-trisubstituted phenyl rings and a 1,2,3,5-tetrasubstituted phenyl ring), four methoxyl groups, two phenolic and five alcoholic hydroxyl groups, one furan and one glycerol unit. In addition, an anomeric proton resonated at δ_H_ 4.67 (1H, d, *J* = 7.8 Hz) and the large coupling constant indicated β-glucosidic linkage. Detailed inspection of the NMR data revealed that the structure of compound **2** was similar to hedyotol C 4″-*O*-β-d-glucopyranoside [20], except that the glucosyl unit was connected to C-7″ in **2** rather than to C-4″, as indicated by the HMBC correlations from H-1″′ (δ_H_ 4.67) to C-7″ (δ_C_ 82.3) and H-7″ (δ_H_ 5.15) to C-1″′ (δ_C_ 104.4). The nature of sugar in compound **2** was further demonstrated to be d-glucose by GC analysis of an acid-treated hydrolysate, as well as comparison of their ^13^C-NMR data with that in the literature [20]. The relative configuration of compound **2** was determined by interpretation of the NOESY spectrum and the chemical shift ratio of C-7″:C-8″ [21]. In the NOESY spectrum of compound **2** (See Figure S13), the correlation of H-7″ (δ_H_ 5.15) with H-8″ (δ_H_ 4.42–4.44) was observed, suggestive of the same α-orientation. Moreover, based on the similar chemical shift ratio of C-7″:C-8″ (δ_C_ 82.3:86.6) to those of hedyotol C 4″-*O-*β-d-glucopyranoside [20], the relative configuration of compound **2** was proposed as an *erythro* configuration. Therefore, compound **2** was established as hedyotol C 7″-*O*-β-d-glucopyranoside.

Compound **3** (Figure 1) was determined to have the same molecular formula C_37_H_46_O_16_ as compound **2** by HR-ESI-MS (*m*/*z* 769.2662 [M + Na]^+^). Their ^1^H- and ^13^C-NMR spectra closely resembled each other (Table 2). The above evidence suggests that compounds **2** and **3** might be stereoisomeric, which could be clarified in accordance with the NOESY spectrum and the chemical shift ratio of C-7″:C-8″ [21]. The differences between compounds **2** and **3** were the absence of NOESY correlations between H-7″ (δ_H_ 5.28) and H-8″ (δ_H_ 4.26–4.27) (See Figure S20) and the similar chemical shift ratio of C-7″:C-8″ (δ_C_ 77.7:86.8) as compared to those of hedyotol D 4″-*O-*β-d-glucopyranoside [20] in compound **3**. This evidence confirmed compounds **2** and **3** were a pair of epimers at C-7″. Accordingly, the relative configuration of compound **3** was presumed as a *threo* configuration. Consequently, compound **3** was identified as hedyotol D 7″-*O-*β-d-glucopyranoside.

Nine known compounds were identified as machicendonal (compound **4**) [22], (7*S*,8*R*)-dihydrodehydrodiconiferyl alcohol (compound **5**) [23], kaempferol-3-*O*-β-d-glucopyranoside (compound **6**) [24], potengriffioside A (compound **7**) [25], kaempferol (compound **8**) [24], 5,7,8,3′-tetrahydroxy-4′-methoxyflavone (compound **9**) [26], 5,7,8-trihydroxy-4′-methoxyflavone (compound **10**) [26], hesperidin (compound **11**) [27], and homoeriodictyol-7-*O-*β-d-glucopyranoside (compound **12**) (Figure 1) [28] by comparing the spectroscopic data with those reported in the literature. Compounds **4**, **8**–**12** were isolated from the genus *Helicteres* for the first time.

All the isolated constituents (compounds **1**–**12**) were evaluated for in vitro anti-complementary activity on CP and AP [16]. As shown in Figure 1 and Table 3, compounds **4** and **5** belonged to the benzofuran lignans and possessed the most potent anti-complementary activity with CH_50_ values of 0.040 ± 0.009 and 0.009 ± 0.002 mM and AP_50_ values of 0.105 ± 0.015 and 0.021 ± 0.003 mM, respectively. Seven flavonoids (compounds **6**–**12**) showed anti-complementary activity with CH_50_ and AP_50_ values of 0.143–1 mM and 0.311–1 mM, respectively. Compounds **1**–**3** exhibited moderate activity on CP and weak activity on AP. It was found that flavonoids with 4′-OH (compounds **8** and **12**) showed stronger anti-complementary activity than those with a -OCH_3_ at C-4′ (compounds **9**–**11**). Thus, the 4′-OH appears to be essential for the complementary activity of flavonoids.

**Figure 3:**
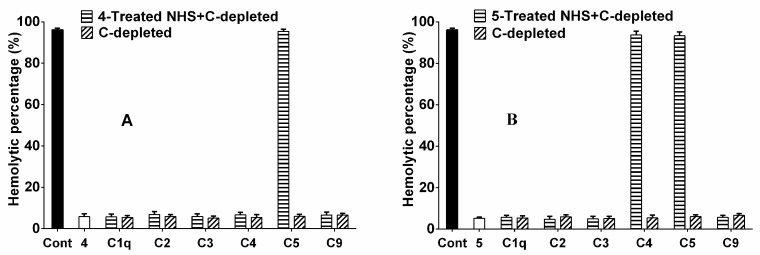
Hemolytic assays of compounds **4** (**A**) and **5** (**B**) for individual components utilizing C-depleted sera.

In order to illuminate the anti-complementary mechanism of compounds **4** and **5**, identification of their targets in the complement activation cascade was conducted using complement-depleted (C-depleted) sera [16]. As shown in Figure 3, compound **4** regained the hemolytic capacity of C5-depleted serum, and compound **5** regained the hemolytic capacity of C4- and C5-depleted sera. These findings suggested that compound **4** probably acted on C1q, C2, C3, C4 and C9, while compound **5** interacted with the C1q, C2, C3 and C9 components of the complement. These results indicate that different compounds can act on different targets in the complement activation cascade. Thus, compounds **4** and **5** are promising candidates for development as anti-complementary agents from *H. angustifolia*. Moreover, the relevant pharmacology and toxicology of these compounds need further investigations.

## 3. Materials and Methods

### 3.1. General Experimental Procedures

The 1D and 2D NMR spectra were recorded on a Bruker Ultrashield Plus 600 MHz spectrometer (Bruker BioSpin Corporation, Billerica, MA, USA). Electrospray ionization mass spectrometry (ESI-MS) spectra were recorded on an Agilent SL G1946D single quadrupole mass spectrometer (Agilent, Foster, CA, USA). High-resolution electrospray ionization mass spectra (HR-ESI-MS) were determined on a Bruker micro time of flight (TOF) spectrometer (Bruker Daltonics Corporation, Billerica, MA, USA). The infrared (IR) spectra were measured on a Thermo Nicolet Avatar 360 spectrophotometer (Thermo Nicolet, Madison, WI, USA). Ultraviolet (UV) spectra were obtained on a Lambda 25 spectrometer (PerkinElmer, Wellesley, MA, USA). The optical rotations were measured on a JASCO P-1020 polarimeter (JASCO Corporation, Tokyo, Japan). The melting point was measured on a micromelting point apparatus which was uncorrected (Yuhua Instruments Co., Ltd., Gongyi, China). Gas chromatography (GC) was carried out on a Shimadzu GC-MS QP 2010 Ultra (Shimadzu Corporation, Kyoto, Japan). Semipreparative high performance liquid chromatography (Semi-HPLC) was run on an Agilent 1100 series (Agilent Technologies, Waldbronn, Germany), equipped with a DAD detector and an ODS column (250 × 10.0 mm, 5 μm, Phenomenex Luna C_18_). Medium pressure liquid chromatography (MPLC) was conducted on a MITSUBISHI GOT 1000 chromatographic instrument (Lisure Science (Suzhou) Co., Ltd., Suzhou, China) with a Santai ILOK ODS column (390 mm × 31.2 mm i.d.). Flash column chromatography was operated by use of normal-phase silica gel (200–300 mesh, Qingdao Marine Chemical Factory, Qingdao, China) and gel Sephadex LH-20 (Pharmacia Fine Chemical Co. Ltd., Uppsala, Sweden). Anti-complement 1q ((Anti-C1q), Human (Goat)), Anti-C2 (Human (Goat)) and Anti-C9 (Human (Goat)) were purchased from Calbiochem (Merck KGaA, Darmstadt, Germany), and Anti-C3 (Human (Goat)), Anti-C4 (Human (Goat)) and Anti-C5 (Human (Goat)) were obtained from Zhejiang Nanfang Reagent Factory, China. Heparin sodium salt (≥150 IU/mg, dry basis) was purchased from Shanghai Aizite Biotech Co. Ltd., China.

### 3.2. Plant Material

Dried aerial parts of *H. angustifolia* were purchased from Nanning, Guangxi Zhuang Autonomous Region of China in March 2011, and authenticated by Prof. Dao-Feng Chen at Fudan University. A voucher specimen (DFC-SZM20110812) has been deposited in the Herbarium of Materia Medica, Department of Pharmacognosy, School of Pharmacy, Fudan University, Shanghai, China.

### 3.3. Extraction and Isolation

The extraction of the dried aerial parts of *H. angustifolia* with 95% ethanol was partitioned successively with petroleum ether, EtOAc, and *n*-BuOH. The EtOAc fraction was selected to further purification due to its significant anti-complementary activity with CH_50_ value of 0.15 ± 0.02 and AP_50_ value of 0.29 ± 0.03 mg/mL. The EtOAc extract (160 g) was subjected to a silica gel column (12 × 50 cm), eluted with CH_2_Cl_2_–MeOH (50:1, 30:1, 20:1, 10:1, 8:1, 5:1, 3:1, 2:1, 1:1, 1:2, and 0:1, *v*/*v*). The CH_2_Cl_2_–MeOH (20:1) fraction (30 g) was subsequently chromatographed over a silica gel column (10 × 40 cm), eluted with CH_2_Cl_2_–MeOH (30:1, 20:1, 10:1, 5:1, 1:1, and 1:2, *v*/*v*) to afford six fractions. Fraction 1 (3.2 g) was further separated by MPLC eluted with MeOH–H_2_O (10:90 to 90:10, *v*/*v*) at 25 mL/min to yield four subfractions (1-A to 1-D). Subfraction 1-B (180 mg) was purified by semipreparative HPLC (MeOH–H_2_O, 42:58, *v/v*) at 3 mL/min to give compounds **4** (6 mg, t_R_ 28.35 min) and **6** (28 mg, t_R_ 35.65 min). Subfraction 1-C (330 mg) was applied to semipreparative HPLC (MeOH–H_2_O, 53:47, *v*/*v*) at 3 mL/min to afford compounds **11** (25 mg, t_R_ 27.34 min), **1** (12 mg, t_R_ 38.73 min), and **8** (17 mg, t_R_ 45.45 min). Subfraction 1-D (550 mg) was separated on a Sephadex LH-20 gel column (2.5 × 100 cm) using MeOH as the eluent to yield compounds **10** (7 mg) and **9** (11 mg). Fraction 2 (8.2 g) was chromatographed by MPLC eluted with MeOH–H_2_O (20:80 to 80:20, *v*/*v*) at 30 mL/min to afford six subfractions (2-A to 2-F). Subfraction 2-C (800 mg) was separated by semipreparative HPLC, isocratically eluted with MeOH–H_2_O (30:70, *v*/*v*) at 3 mL/min to give compound **12** (7 mg, t_R_ 46.21 min). Subfraction 2-D (550 mg) was further subjected to a silica gel column (3.5 × 30 cm) and eluted with a gradient of CH_2_Cl_2_–MeOH (20:1, 10:1, 5:1, 3:1, and 1:1, *v*/*v*) to yield five subfractions (2-D1 to 2-D5). Subfraction 2-D2 (290 mg) was applied to HPLC (MeOH–H_2_O, 40:60, *v*/*v*) to afford compounds **2** (16 mg, t_R_ 12.33 min) and **3** (11 mg, t_R_ 13.98 min). Subfraction 2-D3 (90 mg) and subfraction 2-E (1.3 g) were respectively purified by Sephadex LH-20 (2.5 × 100 cm) with MeOH to give compounds **5** (18 mg) and **7** (0.9 g).

### 3.4. Spectroscopic Data

*8-Acetyl-9-hydroxy-3-methoxy-7-methyl-1-phenalenon* (compound **1**): Yellow crystals (CHCl_3_); m.p. 325–330 °C; UV (CHCl_3_): λ_max_ (log ε): 243.3 (4.17), 342.5 (3.91) nm; IR (KBr) ν_max_: 3373 (OH), 2920, 1698 (C=O), 1623, 1574, 1337, 815 cm^−^^1^; ^1^H- and ^13^C-NMR (600 and 150 MHz, C_5_D_5_N) spectroscopic data see Table 1; HR-ESI-MS: *m/z* 283.0962 [M + H]^+^ (calcd. for C_17_H_15_O_4_^+^; 283.0965).

*Hedyotol C 7″-O-β-d-glucopyranoside* (compound **2**): White amorphous powders; [α]${}_{D}^{25}$ +14.25 (*c* 0.4, MeOH); UV (MeOH): λ_max_ (log ε): 218.0 (3.83), 279.7 (3.15) nm; IR (KBr) ν_max_: 3419 (OH), 2936, 1594, 1512, 1457, 1276, 1117 cm^−^^1^; ^1^H- and ^13^C-NMR (600 and 150 MHz, CD_3_OD) spectroscopic data see Table 2; HR-ESI-MS: *m/z* 769.2662 [M + Na]^+^ (calcd. for C_37_H_46_NaO_16_^+^; 769.2678).

*Hedyotol D 7″-O-β-d-glucopyranoside* (compound **3**): White amorphous powders; [α]${}_{D}^{25}$ –13.50 (*c* 0.3, MeOH); UV (MeOH): λ_max_ (log ε): 214.0 (3.93), 279.6 (3.22) nm; IR (KBr) ν_max_: 3419 (OH), 2926, 1600, 1506, 1463, 1123 cm^−^^1^; ^1^H- and ^13^C-NMR (600 and 150 MHz, CD_3_OD) spectroscopic data see Table 2; HR-ESI-MS: *m*/*z* 769.2661 [M + Na]^+^ (calcd. for C_37_H_46_NaO_16_^+^; 769.2678).

### 3.5. Acid Hydrolysis of Compounds ***2*** and ***3***

The acid hydrolysis was performed according to the modified method of Song et al. [29]. Compound **2** (2 mg) was dissolved in 2 mol/L HCl (1 mL), and heated at 95 °C for 2 h. After hydrolysis, the reaction mixture was extracted with EtOAc and H_2_O, and the aqueous layer was neutralized with silver carbonate (10 mg). Then the supernatant was concentrated to dryness and further evaporated to dryness under N_2_ for 2 h. Subsequently, the residue was stirred with l-cysteine methyl ester hydrochloride (2 mg) in anhydrous pyridine (0.2 mL) at 60 °C for 1 h. The dried reactant was fractionalized with H_2_O and *n*-hexane (each 0.5 mL). The *n*-hexane layer was collected and subjected to GC-MS. The acid hydrolysis of compound **3** was conducted in the same procedure as compound **2**. The GC conditions were: column temperature 150 °C, injector temperature 270 °C, carrier gas N_2._ d-glucose was detected from compounds **2** and **3** (t_R_ 15.523 and 15.524 min) by comparison with authentic samples: t_R_d-glucose 15.513 min, and l-glucose 15.808 min.

### 3.6. Anti-Complementary Activity Assay Against CP and AP

The anti-complementary activities against the CP and AP were investigated with the method of Xu et al. [16]. Heparin was used as positive control drug. The results of anti-complementary activity of compounds **1**–**12** are displayed in Table 3. Anti-complement activity was determined as the mean of triplicate measurements at each concentration and expressed as 50% inhibitory concentration (CH_50_ and AP_50_ values). All results were performed statistical analysis to compare the CH_50_ and AP_50_ values between each compound and positive control with unpaired *t* test.

### 3.7. Identification of the Targets in the Complement Activation Cascade

Assay to identify the targets in the complement activation cascade was performed according to the method of Di et al. [30]. The results of compounds **4** and **5** were displayed in Figure 3. Data were expressed as mean ± SD of triplicate measurements.

## 4. Conclusions

A first phenalenon derivative (compound **1**) with an acetyl side chain at C-8 and a pair of new sesquilignan epimers (compounds **2** and **3**) with a glucosyl unit at C-7**″** together with nine known compounds were isolated from the aerial parts of *H. angustifolia*. Chemical structures of the isolated compounds were identified on the basis of extensive spectroscopic data. Compounds **4**, **8**–**12** were isolated for the first time from the aerial parts of *H. angustifolia*. Eleven compounds exhibited anti-complementary activity, and the targets of two compounds (**4** and **5**) in the complement activation cascade were also identified. In conclusion, the anti-complementary constituents of *H. angustifolia* possessed chemical diversity, and consisted mostly of flavonoids and lignans in this study.

## Figures and Tables

**Table 1:** ^1^H- and ^13^C-NMR (600 and 150 MHz) data of compound **1** in C_5_D_5_N, δ in ppm, *J* in Hz.

No.	δ_H_	δ_C_	No.	δ_H_	δ_C_
1		181.1	7		144.1
2	6.66 s	100.2	8		136.3
3		166.6	9		174.2
3a		120.8	9a		106.5
3b		127.1	3-OCH_3_	3.92 s	56.4
4	8.43 d (7.8)	127.9	7-CH_3_	2.58 s	16.0
5	7.58 t (7.8)	123.7	8-COCH_3_		204.1 (CO)
6	8.24 d (7.8)	130.8		2.72 s	32.1 (CH_3_)
6a		125.4	9-OH	17.36 s	

**Table 2:** ^1^H- and ^13^C-NMR (600 and 150 MHz) data for compounds **2** and **3** in CD_3_OD, δ in ppm, *J* in Hz.

No.	2		3	
	δ_H_	δ_C_	δ_H_	δ_C_
1		133.7		133.7
2	6.97 d (1.8)	111.0	6.97 d (1.4)	111.0
3		149.1		149.1
4		147.4 ^a^		147.4 ^a^
5	6.79 d (8.1)	116.1	6.79 d (8.1)	116.1
6	6.83 dd (8.1, 1.7)	120.1	6.83 dd (8.0, 1.3)	120.1
7	4.72 d (4.8)	87.5	4.73 d (3.6)	87.5
8	3.14 ddd (11.3, 8.3, 4.9)	55.3 ^b^	3.13–3.15 overlapped	55.3 ^b^
9	4.24–4.30 overlapped, 3.88–3.91 overlapped	72.9 ^c^	4.27–4.31 overlapped, 3.88–3.91 overlapped	72.9 ^c^
1′		138.9		138.9
2′,6′	6.64 s	104.2	6.66 s	104.1
3′,5′		154.3		154.5
4′		136.2		136.4
7′	4.75 d (4.6)	87.2	4.76 d (3.0)	87.3
8′	3.14 ddd (11.3, 8.3, 4.9)	55.7 ^b^	3.13–3.15 overlapped	55.7 ^b^
9′	4.24–4.30 overlapped, 3.88–3.91 overlapped	72.8 ^c^	4.27–4.31 overlapped, 3.88–3.91 overlapped	72.7 ^c^
1′′		132.4		130.8
2′′	7.03 d (1.7)	112.7	7.24 brs	113.1
3′′		148.4		148.7
4′′		147.1 ^a^		147.1 ^a^
5′′	6.72 d (8.1)	115.3	6.80 d (8.0)	115.5
6′′	6.87 dd (8.1, 1.7)	121.9	6.92 dd (8.0, 1.3)	122.0
7′′	5.15 d (6.2)	82.3	5.28 d (3.2)	77.7
8′′	4.42–4.44 m	86.6	4.26–4.27 m	86.8
9′′	4.05 dd (12.4, 4.0) 3.76 dd (12.4, 3.0)	61.5	3.88–3.91 overlapped 3.44 dd (11.4, 4.5)	61.4
β-d-glc				
1′′′	4.67 d (7.8)	104.4	4.21 d (7.5)	101.0
2′′′	3.27-3.29 m	75.7	3.33–3.34 m	75.2
3′′′	3.38 d (9.0)	78.1	3.28–3.31 overlapped	77.7
4′′′	3.30–3.32 m	71.5	3.28–3.31 overlapped	71.9
5′′′	3.19 ddd (9.6, 5.4, 2.5)	77.8	3.12–3.13 m	77.8
6′′′	3.73 dd (11.9, 2.3) 3.60 dd (11.8, 5.4)	62.7	3.83–3.85 m 3.69 dd (11.9, 5.9)	62.7
3-OCH_3_	3.87 s	56.41 ^d^	3.88 s	56.37 ^d^
3′,5′-OCH_3_	3.80 s	56.6	3.74 s	56.5
3′′-OCH_3_	3.83 s	56.43 ^d^	3.85 s	56.42 ^d^

^a−d^ The assignments in each column may be interchanged.

**Table 3:** Anti-complementary activity of compounds **1**–**12** against classical and alternative pathways.

Compound	CH_50_ (mM) ^a^	AP_50_ (mM) ^a^
**1**	0.744 ± 0.099	>1
**2**	0.419 ± 0.043	>1
**3**	0.249 ± 0.021	>1
**4**	0.040 ± 0.009	0.105 ± 0.015
**5**	0.009 ± 0.002 **^,b^	0.021 ± 0.003 **^,b^
**6**	0.877 ± 0.081	>1
**7**	0.143 ± 0.019	0.335 ± 0.040
**8**	0.147 ± 0.022	0.311 ± 0.033
**9**	0.232 ± 0.25	0.501 ± 0.065
**10**	0.511 ± 0.043	0.984 ± 0.107
**11**	>1	>1
**12**	0.351 ± 0.033	0.556 ± 0.061
**Heparin ^c^**	0.026 ± 0.005	0.055 ± 0.008

^a^ Data were represented as mean ± SD of three independent measurements (*n* = 3), and described as 50% hemolytic inhibitory concentration (CH_50_ for classical pathway and AP_50_ for alternative pathway). **^,b^ *p* < 0.01, compared to the positive control group. ^c^ Heparin was used as the positive control (mg/mL).

## Supplementary Materials

The 1D and 2D NMR spectra for compounds **1**–**3** are available as supporting data online at http://www.mdpi.com/1420-3049/21/11/1506/s1.

## References

[1] Carroll M.C., Fisher M.B. (1997). Complement and the immune response. Curr. Opin. Immunol..

[2] Morgan B.P., Harris C.L. (2003). Complement therapeutics; history and current progress. Mol. Immunol..

[3] Zhu H.W., Di H.Y., Zhang Y.Y., Zhang J.W., Chen D.F. (2009). A protein-bond polysaccharide from the stem bark of *Eucommia ulmoides* and its anti-complementary effect. Carbohydr. Res..

[4] Mollnes T.E., Song W.C., Lambris J.D. (2002). Complement in inflammatory tissue damage and disease. Trends Immunol..

[5] Vovel C.W., Fritzinger D.C. (2010). Cobra venom factor: Structure, function, and humanization for therapeutic complement depletion. Toxicon.

[6] Thorgersen E.B., Ghebremariam Y.T., Thurman J.M., Fung M., Nielsen E.W., Holers V.M., Kotwal G.J., Mollnes T.E. (2007). Candidate inhibitors of porcine complement. Mol. Immunol..

[7] Qu H.C., Ricklin D., Lambris J.D. (2009). Recent developments in low molecular weight complement inhibitors. Mol. Immunol..

[8] Risitano A.M. (2015). Current and future pharmacologic complement inhibitors. Hematol/Oncol. Clin. N. Am..

[9] Jiangsu New Medical College (1996). Dictionary of Chinese Herb Medicines.

[10] Chen W.L., Tang W.D., Lou L.G., Zhao W.M. (2006). Pregnane, coumarin and lupane derivatives and cyto toxic constituents from *Helicters angustifolia*. Phytochemistry.

[11] Pan M.H., Chen C.M., Lee S.W., Chen Z.T. (2008). Cytotoxic triterpenoids from the root bark of *Helicters angustifolia*. Chem. Biodivers..

[12] Guo X.D., Huang Z.S., Bao Y.D., An L.K., Ma L., Gu L.Q. (2005). Two new sesquiterpenoids from *Helicters angustifolia*. Chin. Chem. Lett..

[13] Chen M., Muchersie E., Luo C., Forrester J.V., Xu H.P. (2010). Inhibition of the alternative pathway of complement activation reduces inflammation in experimental autoimmune uveoretinitis. Eur. J. Immunol..

[14] Ignatius A., Schoengraf P., Kreja L., Liedert A., Rechnagel S., Kandert S., Brenner R.E., Schneider M., Lambris J.D. (2011). Complement C3a and C5a modulate osteoclast formation and inflammatory response of osteoblasts in synergism with IL-1β. J. Cell. Biochem..

[15] Zhang T., Chen D.F. (2008). Anticomplementary principles of a Chinese multiherb remedy for the treatment and prevention of SARS. J. Ethnopharmacol..

[16] Xu H., Zhang Y.Y., Zhang J.W., Chen D.F. (2007). Isolation and characterization of an anti-complementary polysaccharide D3-S1 from the roots of *Bupleurum smithii*. Int. Immunopharmacol..

[17] Kobayashi M., Matsumoto T. (1975). Ungewöhnliche wichterle-reaktionen. Eine neue synthese von phenalenonen. Bull. Chem. Soc. Jpn..

[18] Ernst-Russell M.A., Chai C.L.L., Elix J.A., McCarthy P.M. (2000). Myeloconone A2, a new phenalenone from the lichen *Myeloconis erumpens*. Aust. J. Chem..

[19] Tohru K., Satoko M., Shigetoshi K., Takaaki T. (1985). Studies on the constituents of medicinal and related plants in Sri Lanka. III. Novel sesquilignans from *Hedyotis lawsoniae*. Chem. Pharm. Bull..

[20] Yukinori M., Tsuyoshi T. (1998). Studies on constituents of *Scutellaria* species X IX: lignan glycosides of roots of *Scutellaria baicalensis* Georgi. Nat. Med..

[21] Bardet M., Robert D., Lundquist K., Von-Unge S. (1998). Distribution of erythro and threo forms of different types of β-O-4 structures in aspen lignin by carbon-13 NMR using the 2D INADEQUATE experiment. Magn. Reson. Chem..

[22] Talaptra B., Ray T., Talapatra S.K. (1978). Structures of three new norlignans from *Machilus glaucescens* and their partial syntheses. Chem. Nat. Prod..

[23] Kuang H., Xia Y., Yang B., Wang Q., Lu S. (2009). Lignan constituents from *Chloranthus japonicus* Sieb.. Arch. Pharm. Res..

[24] Yi Y., Wu X., Wang Y., Ye W.C., Zhang Q.W. (2011). Studies on the flavonoids from the flowers of *Hylocereus undatus*. J. Chin. Med. Mater..

[25] Zhong H.J., Chen J.J., Wang H.Y., Luo S.D. (2000). Chemical investigation on *Potentilla griffithii* var. *velutina*. Chin. Tradit. Herb. Drugs..

[26] Xia P.F., Song S., Feng Z.M., Zhang P.C. (2009). Chemical constituents from leaves of *Sterculia foetida*. Zhong Guo Zhong Yao Za Zhi.

[27] Yang N.Y., Duan J.A., Li P., Qian S.H. (2006). Studies on flavonoids of *Lysimachia christinae* Hance. Chin. Pharm. J..

[28] Kong D.Y., Luo S.G., Li H.T., Lei X.H. (1987). Studies on chemical components of *Viscum coloratum* L.. Pharm. Ind..

[29] Song W.H., Chen Z.H., Chen D.F. (2014). Anticomplement monoterpenoid glucosides from the root bark of *Paeonia suffruticosa*. J. Nat. Prod..

[30] Di H.Y., Zhang Y.Y., Chen D.F. (2013). An anti-complementary polysaccharide from the roots of *Bupleurum chinense*. Int. J. Biol. Macromol..

