# Supplementary material for: Anti-Complementary Components of Helicteres angustifolia
Source: Molecules. 2016 Nov 10;21(11):1506. doi: 10.3390/molecules21111506 (PMC6273495; doi:10.3390/molecules21111506)
Supplement: Supplementary file 1 [file molecules-21-01506-s001.pdf]

Supplementary Materials: Anti-Complementary Components of *Helicteres angustifolia*

Xiang Yin, Yan Lu, Zhi-Hong Cheng and Dao-Feng Chen

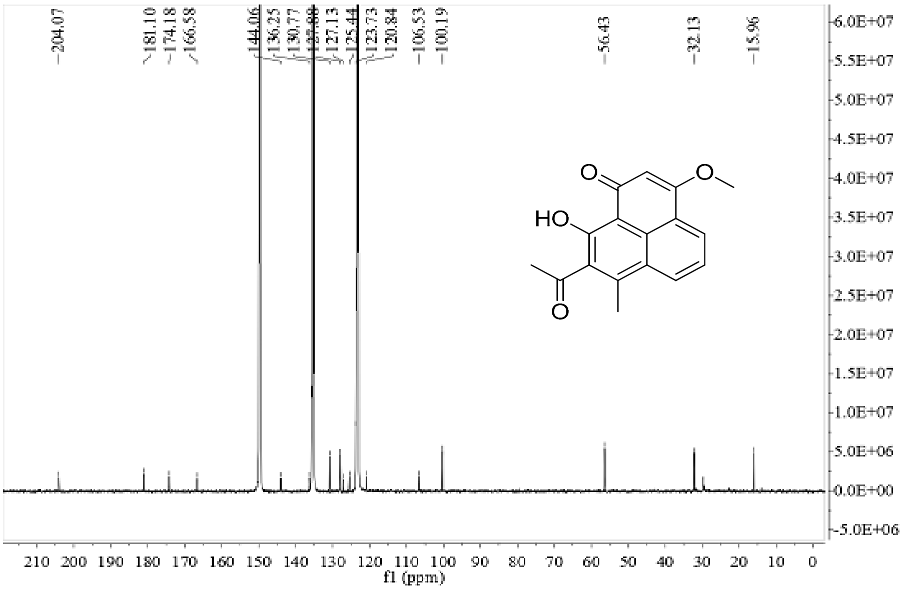

Figure S1. ^{13}C-NMR spectrum of compound **1** in C_5D_5N.

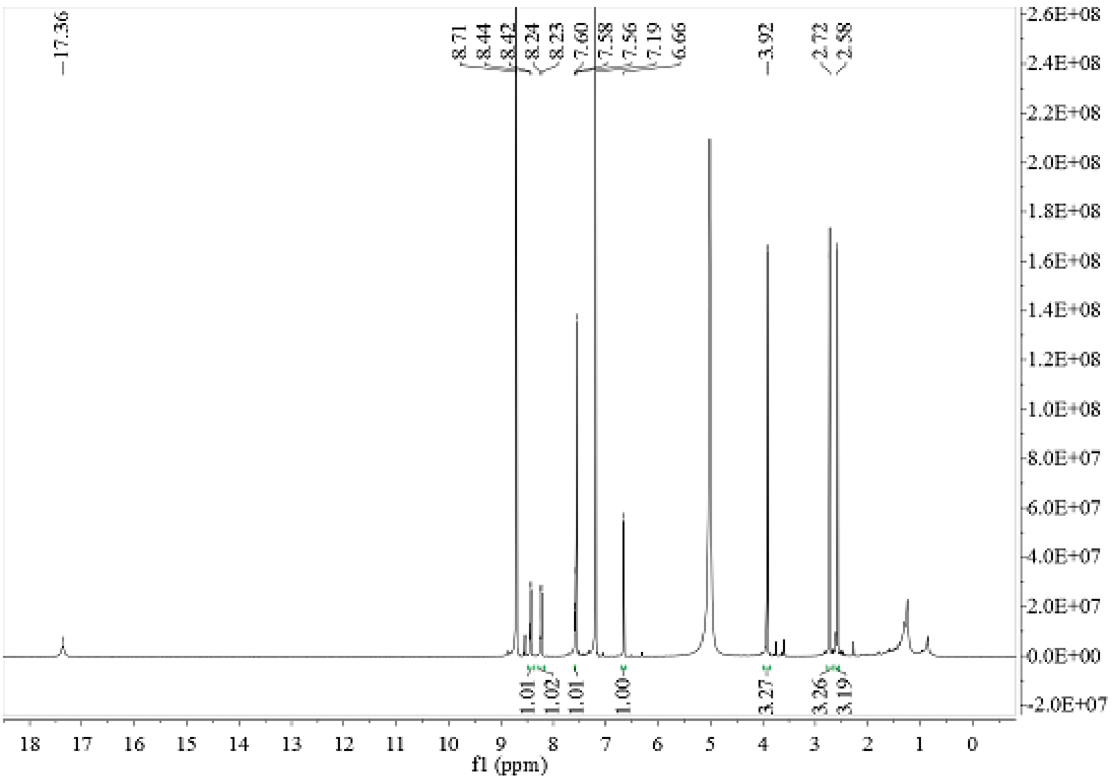

Figure S2. ^{1}H-NMR spectrum of compound **1** in C_5D_5N.

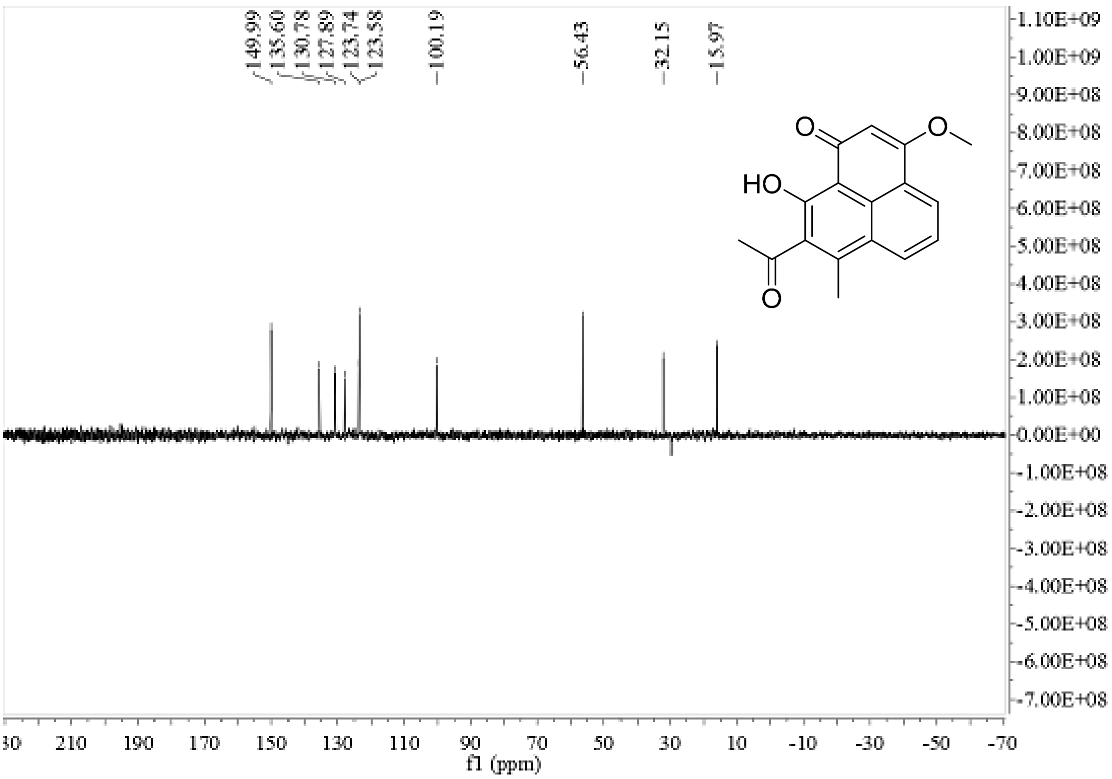

Figure S3. DEPT (135°) spectrum of compound **1** in C_5D_5N.

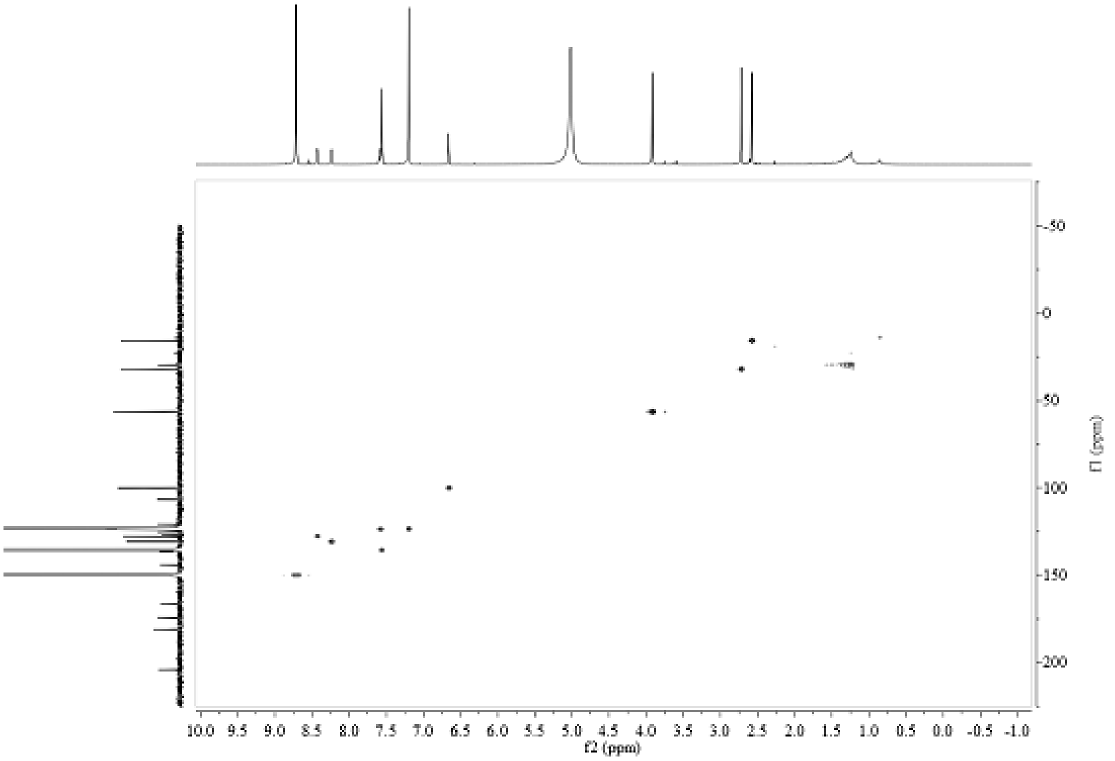

Figure S4. HSQC spectrum of compound **1** in C_5D_5N.

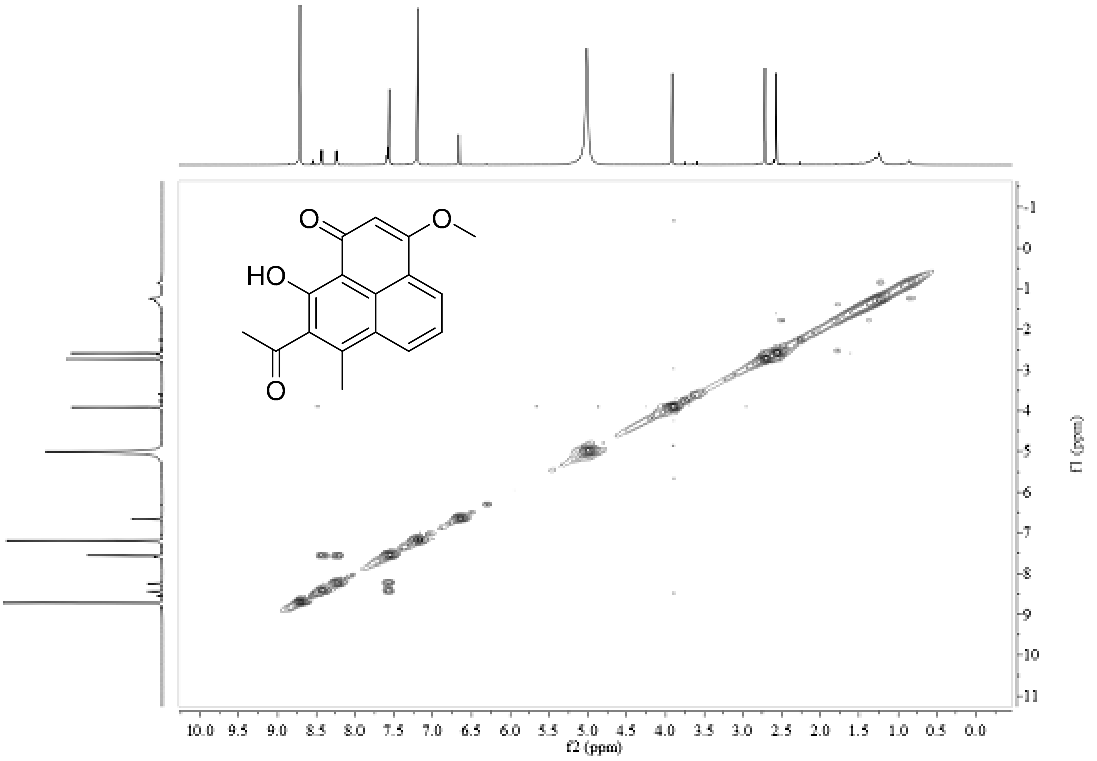

Figure S5. ^{1}H-^{1}H COSY spectrum of compound **1** in C_5D_5N.

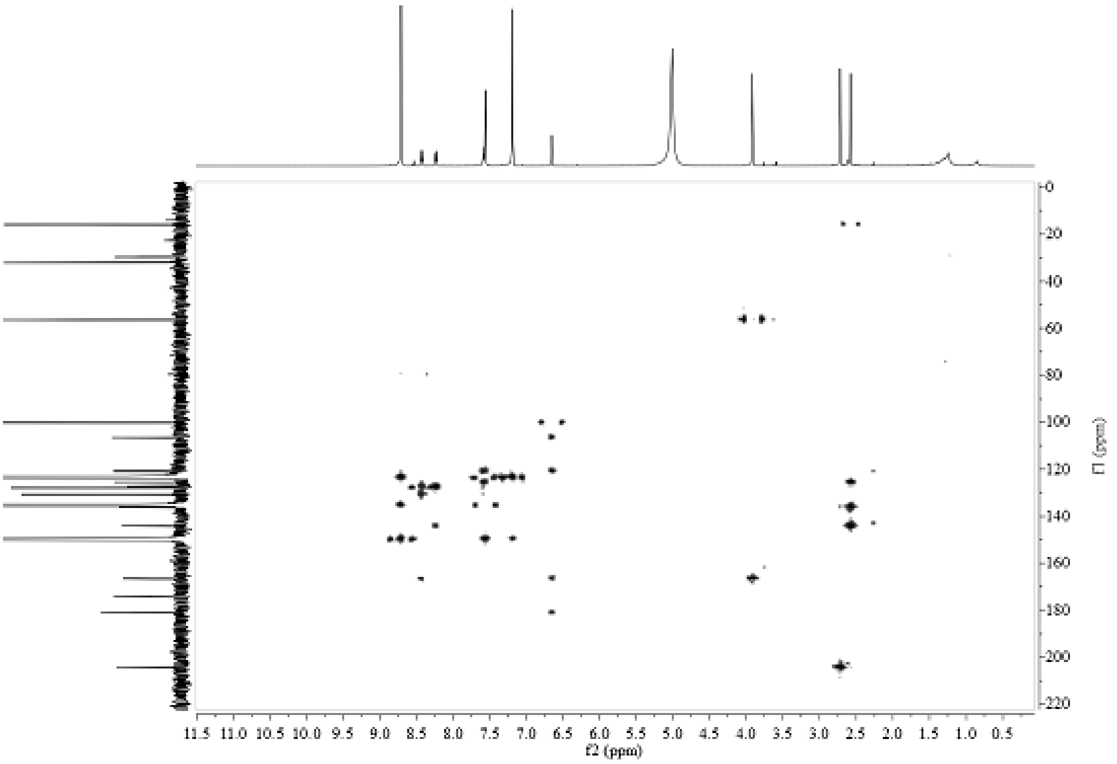

Figure S6. HMBC spectrum of compound **1** in C_5D_5N.

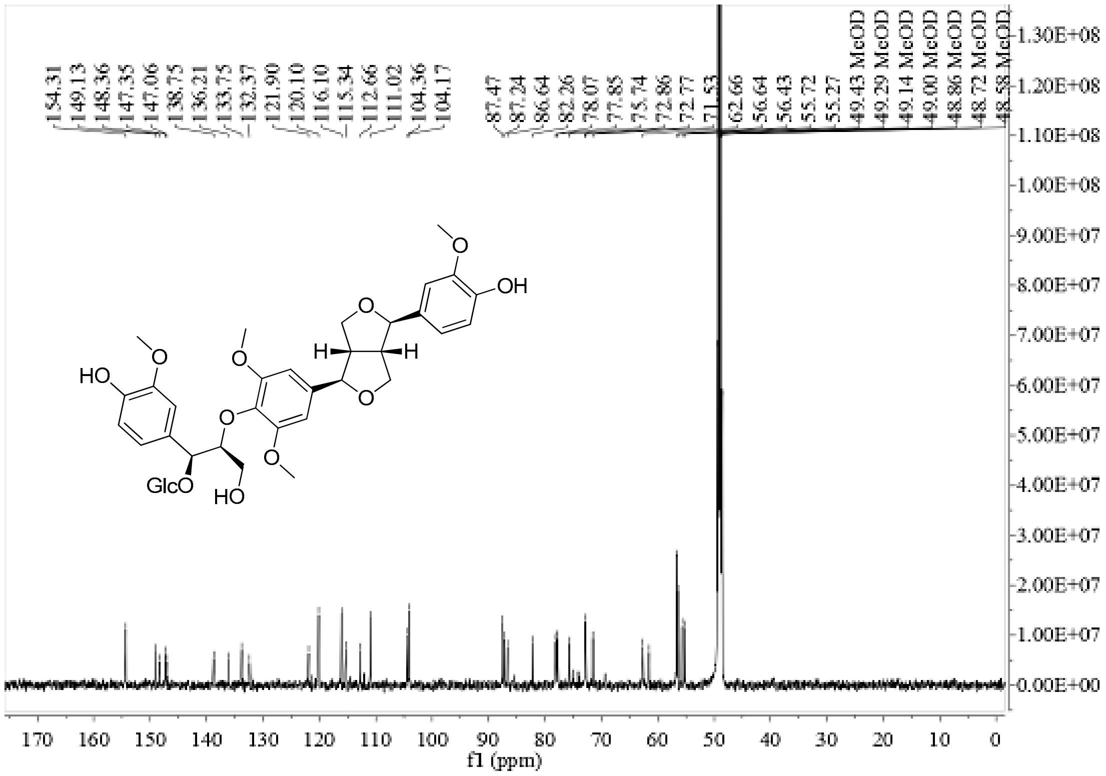

Figure S7. ^{13}C-NMR spectrum of compound **2** in CD_3OD.

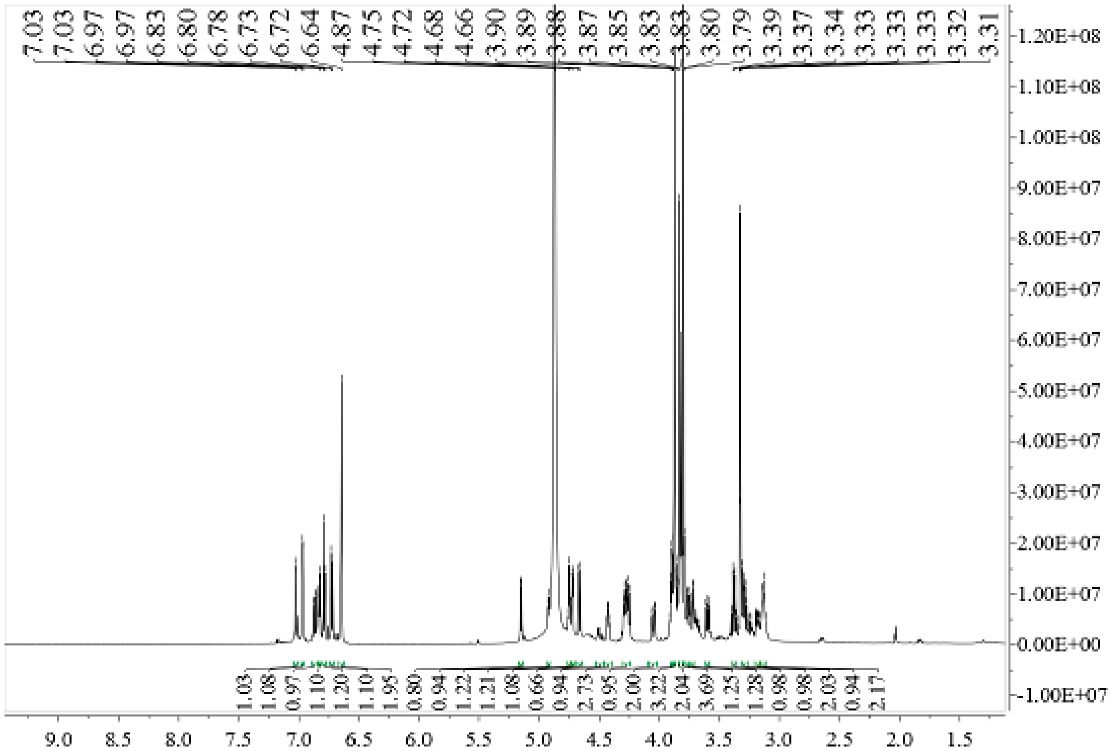

Figure S8. ^{1}H-NMR spectrum of compound **2** in CD_3OD.

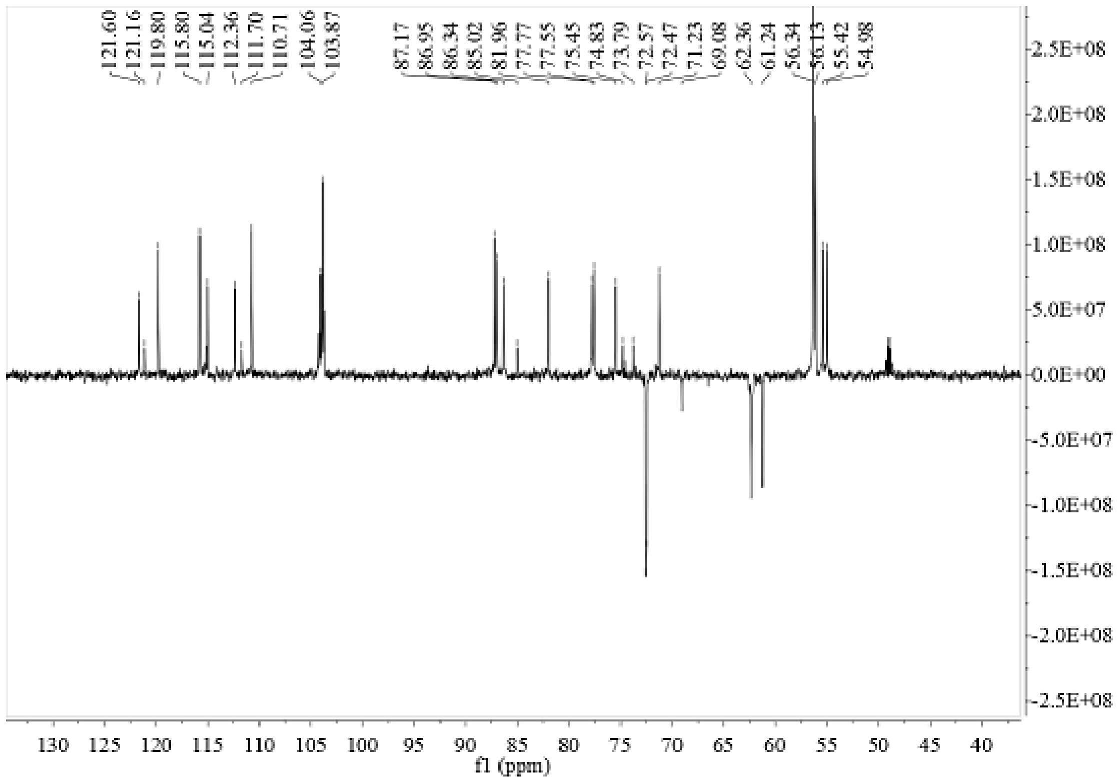

Figure S9. DEPT (135°) spectrum of compound **2** in CD_3OD.

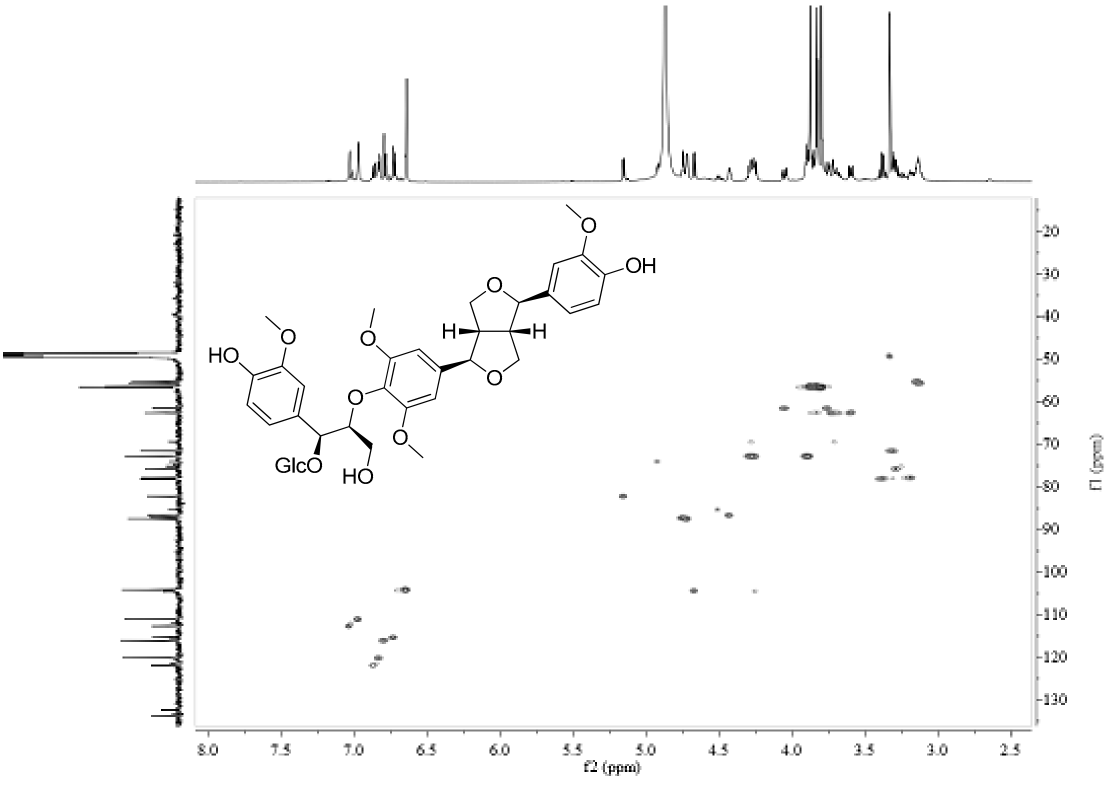

Figure S10. HSQC spectrum of compound **2** in CD_3OD.

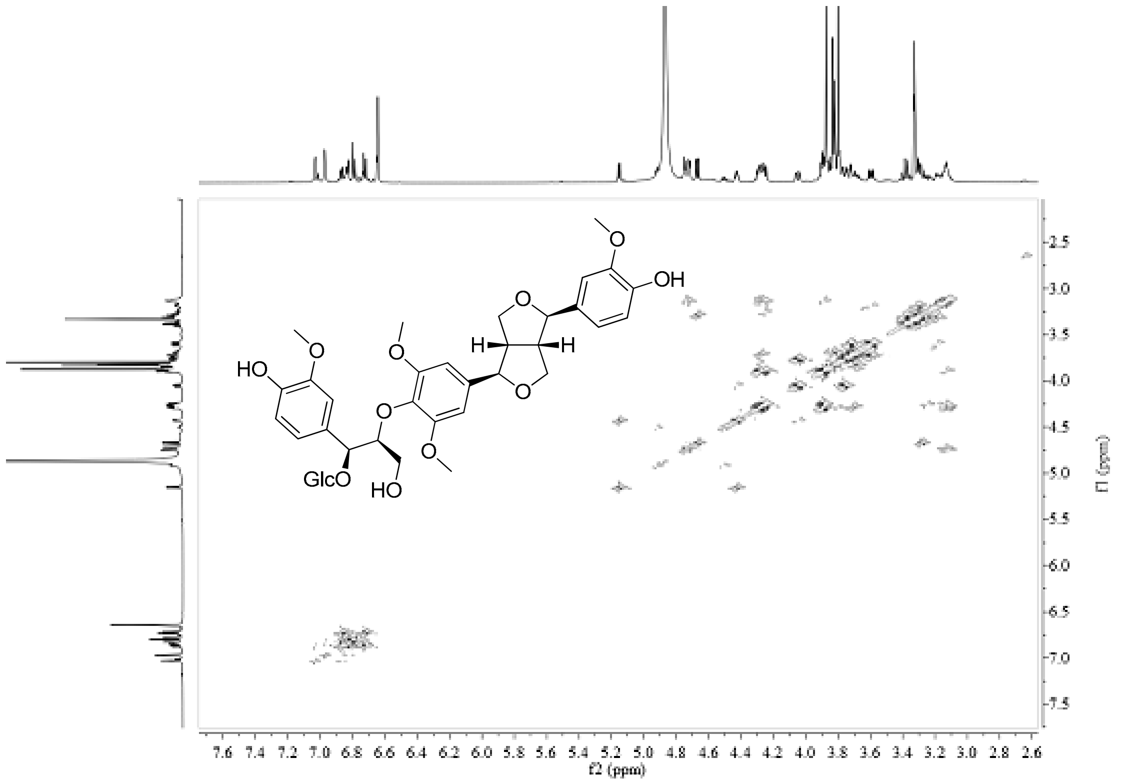

Figure S11. ^{1}H-^{1}H COSY spectrum of compound **2** in CD_3OD.

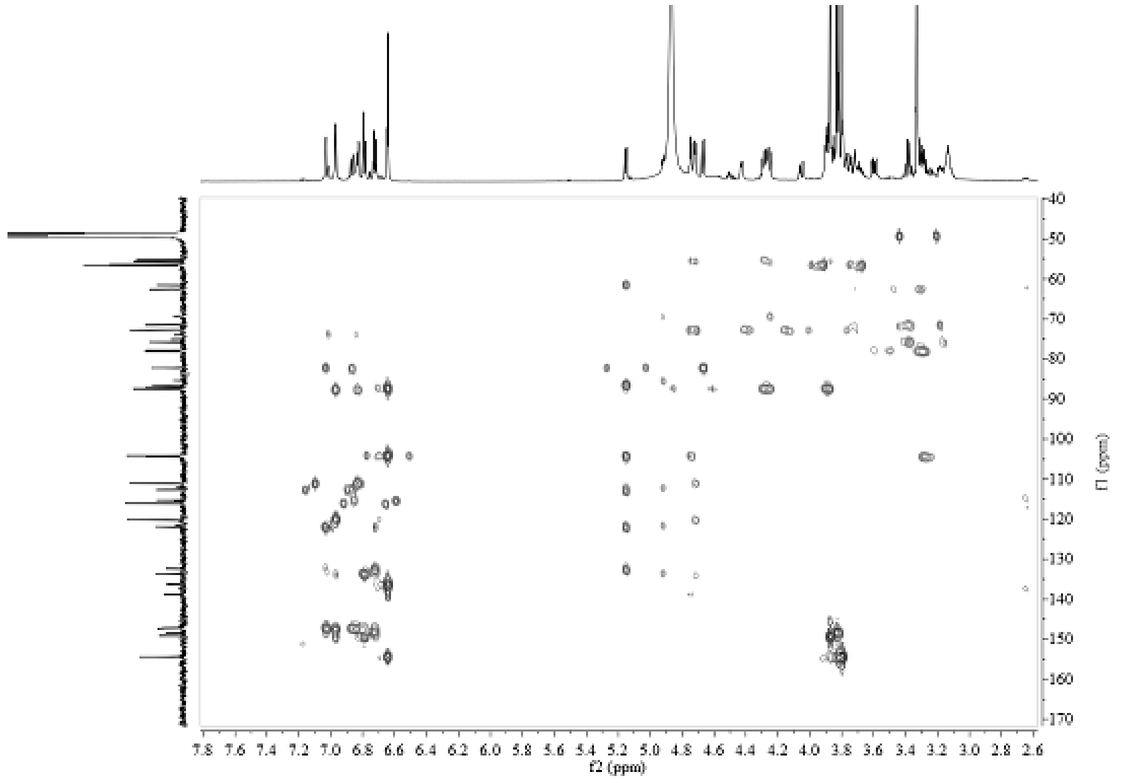

Figure S12. HMBC spectrum of compound **2** in CD_3OD.

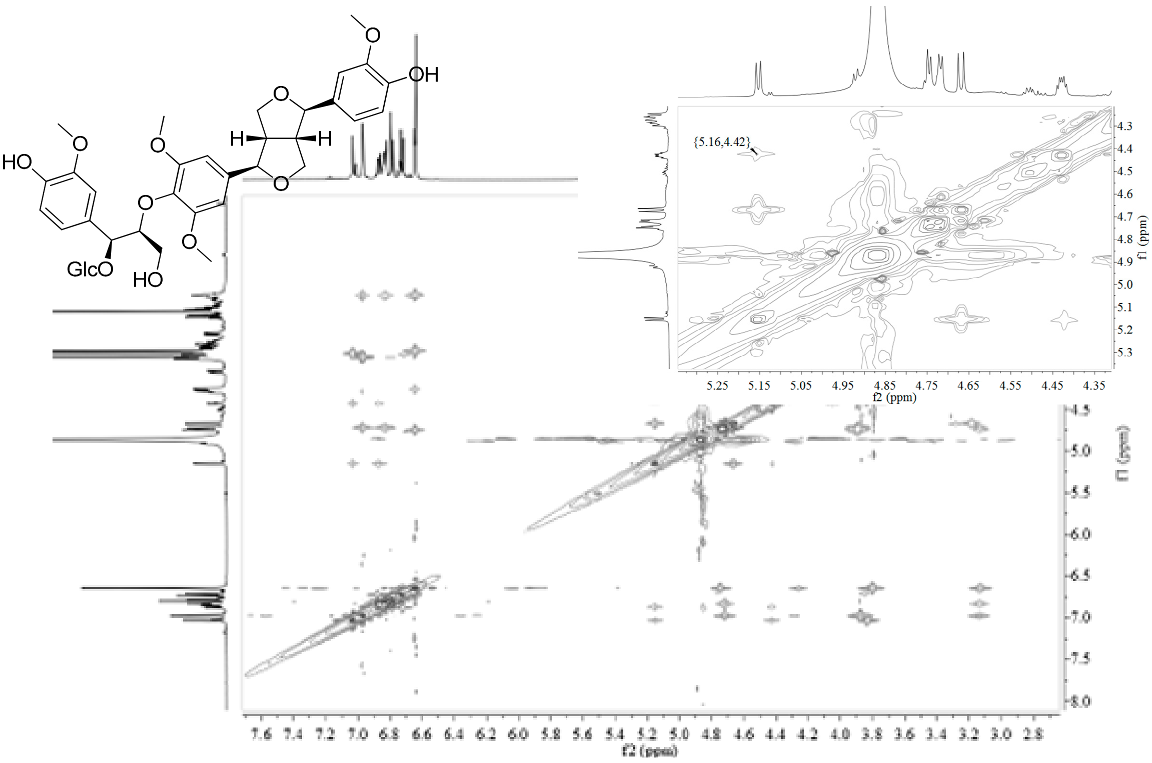

Figure S13. NOESY spectrum of compound **2** in CD_3OD.

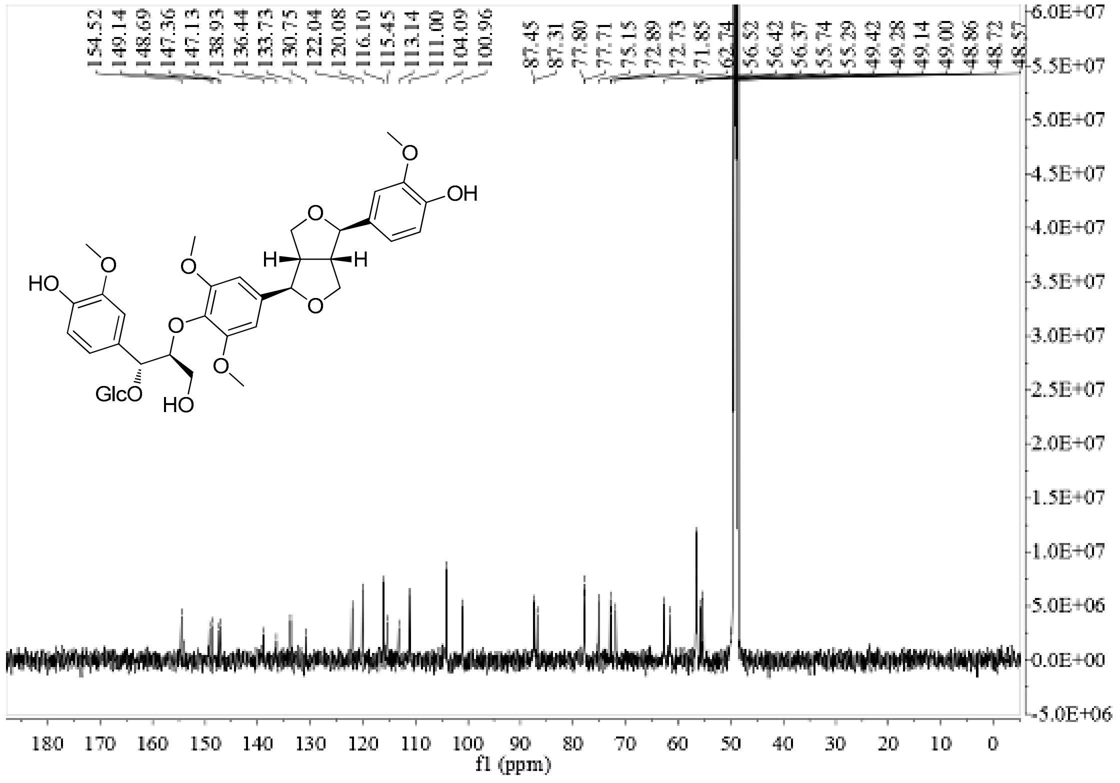

Figure S14. ^{13}C-NMR spectrum of compound **3** in CD_3OD.

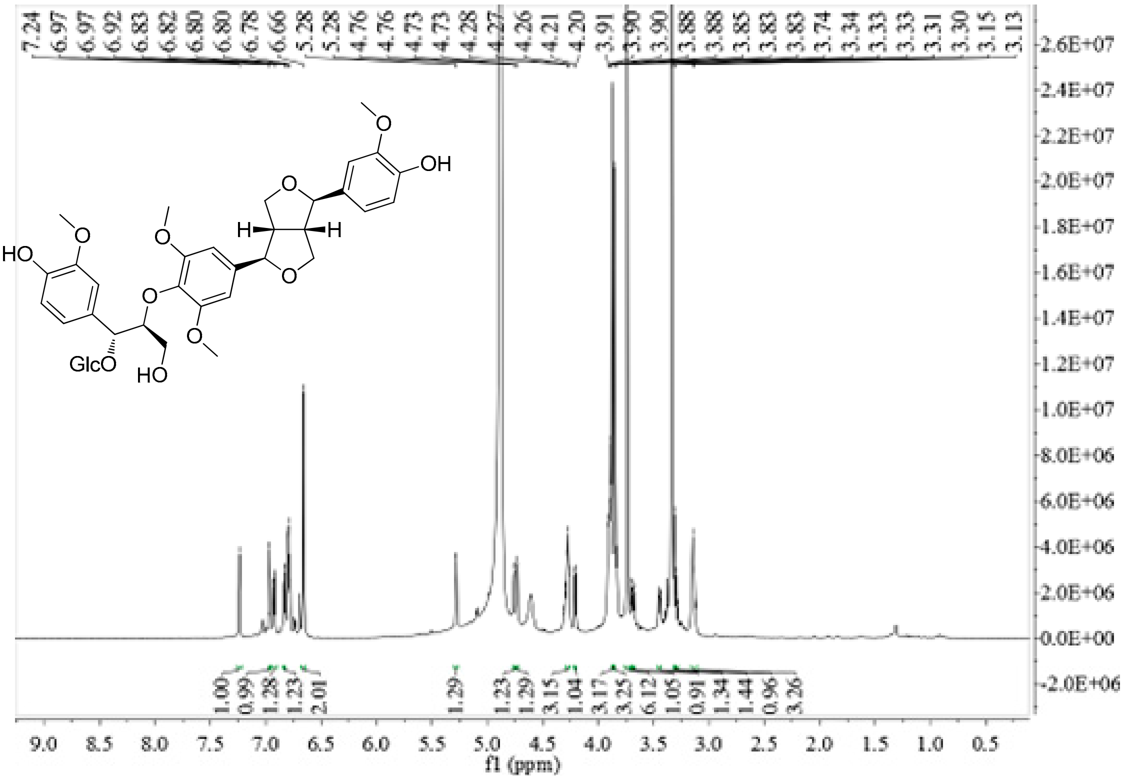

Figure S15. ^{1}H-NMR spectrum of compound **3** in CD_3OD.

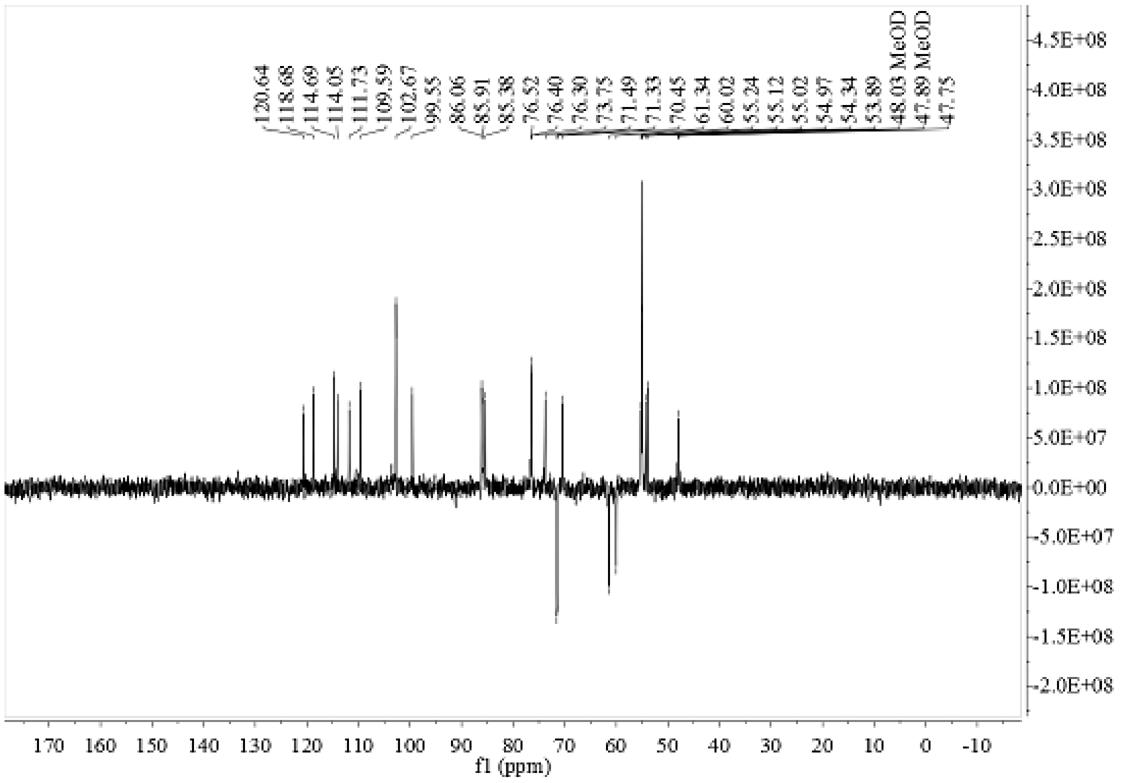

Figure S16. DEPT (135°) spectrum of compound **3** in CD_3OD.

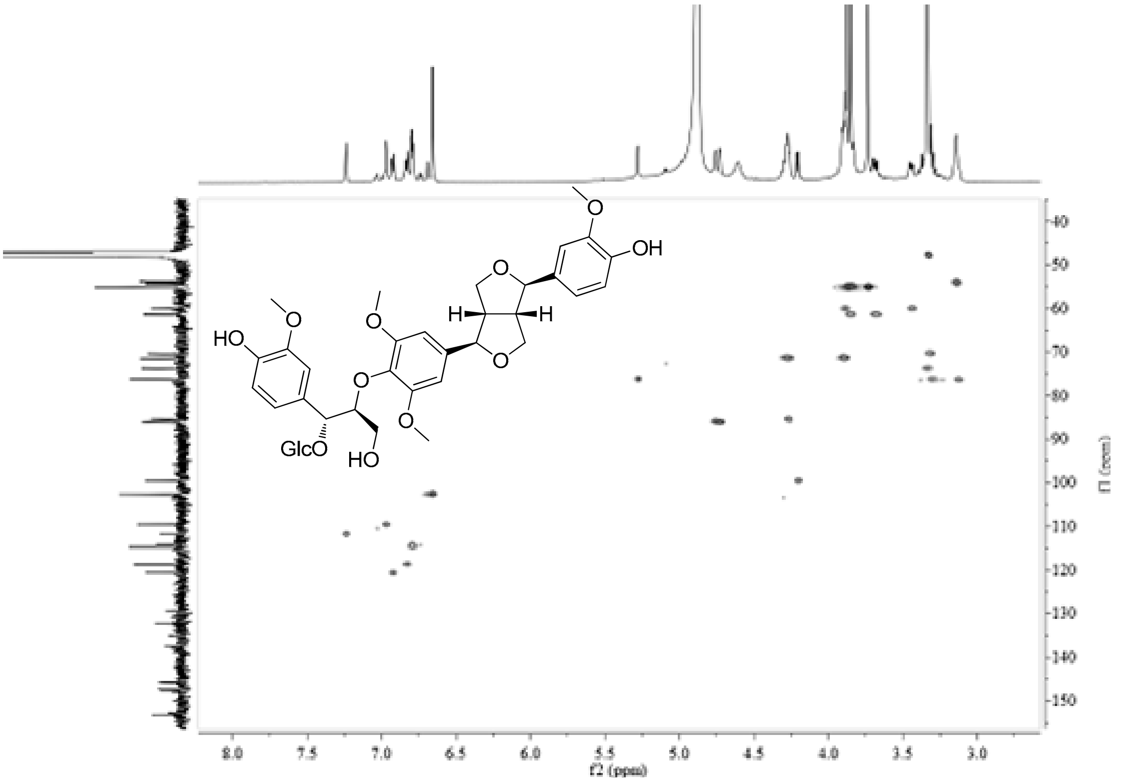

Figure S17. HSQC spectrum of compound **3** in CD_3OD.

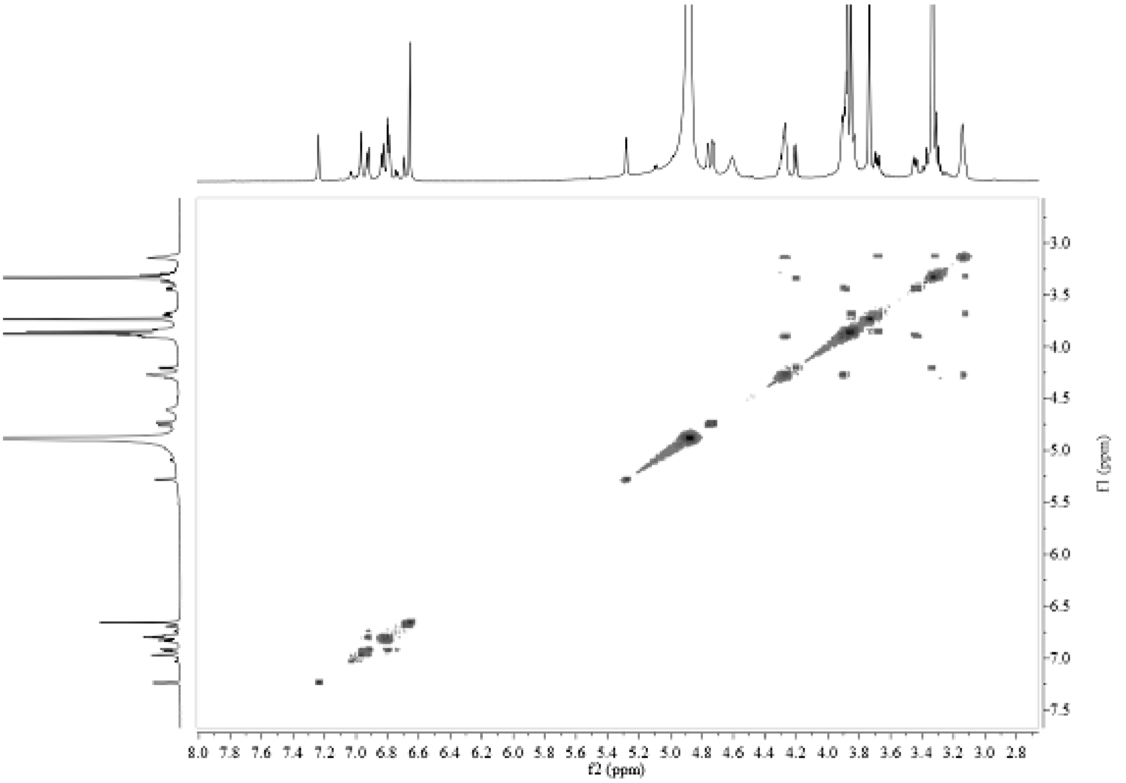

Figure 18S. ^1H-^1H COSY spectrum of compound **3** in CD_3OD.

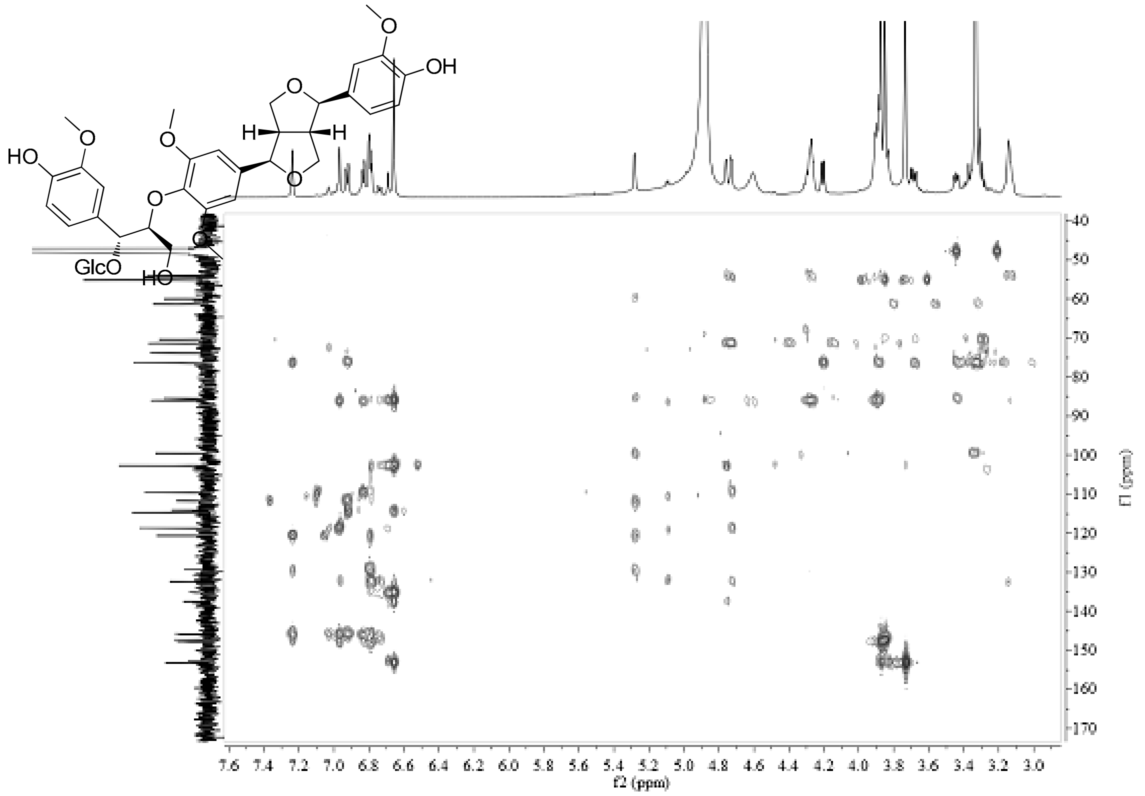

Figure S19. HMBC spectrum of compound **3** in CD_3OD.

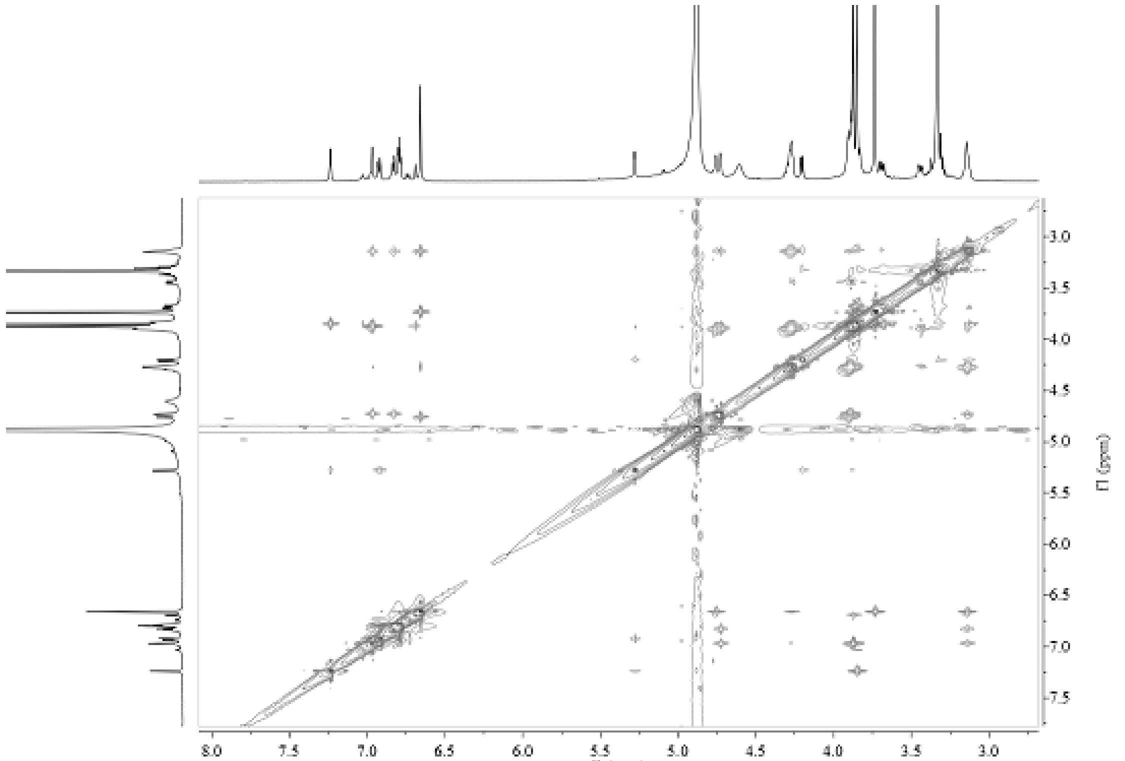

Figure S20. NOESY spectrum of compound **3** in CD_3OD.
